# Explore the expression of mitochondria-related genes to construct prognostic risk model for ovarian cancer and validate it, so as to provide optimized treatment for ovarian cancer

**DOI:** 10.3389/fimmu.2024.1458264

**Published:** 2024-10-16

**Authors:** Zheng Yunyun, Wang Guihu, Jiang An

**Affiliations:** ^1^ Department of Hepatobiliary Pancreas Surgery and Liver Transplantation, The Second Affiliated Hospital, Xi’an Jiaotong University, Xi’an, China; ^2^ Department of Obstetrics and Gynecology, The First Affiliated Hospital of AFM (Air Force Medical University), Xi’an, Shaanxi, China; ^3^ National and Local Joint Engineering Research Center of Biodiagnostics and Biotherapy, Second Affiliated Hospital, Xi’an Jiaotong University, Xi’an, China

**Keywords:** mitochondria, differentially expressed genes, prediction model, ovarian cancer, immunotherapy

## Abstract

**Background:**

The use of gene development data from public database has become a new starting point to explore mitochondrial related gene expression and construct a prognostic prediction model of ovarian cancer.

**Methods:**

Data were obtained from the TCGA and ICGC databases, and the intersection with mitochondrial genes was used to obtain the differentially expressed genes. q-PCR, Cox proportional risk regression, minimal absolute contraction and selection operator regression analysis were performed to construct the prognostic risk model, and ROC curve was used to evaluate the model for centralized verification. The association between risk scores and clinical features, tumor mutation load, immune cell infiltration, macrophage activation analysis, immunotherapy, and chemosensitivity was further evaluated.

**Results:**

A prognostic risk score model for ovarian cancer patients was constructed based on 12 differentially expressed genes. The score was highly correlated with ovarian cancer macrophage infiltration and was a good predictor of the response to immunotherapy. M1 and M2 macrophages in the ovarian tissue in the OV group were significantly activated, providing a reference for the study of the polarity change of tumor-related macrophages for the prognosis and treatment of ovarian cancer. In terms of drug sensitivity, the high-risk group was more sensitive to vinblastine, Acetalax, VX-11e, and PD-0325901, while the low-risk group was more sensitive to Sabutoclax, SB-505124, cisplatin, and erlotinib.

**Conclusion:**

The prognostic risk model of ovarian cancer associated to mitochondrial genes built on the basis of public database better evaluated the prognosis of ovarian cancer patients and guided individual treatment.

## Introduction

Ovarian cancer is one of the three most common gynecological malignancies. The Global Cancer Statistics Report 2020 (GLOBOCAN 2020) (1) released by the International Agency for Research on Cancer shows that the incidence and mortality of ovarian cancer rank third and second, respectively, among gynecological malignancies. The GLOBOCAN 2020 database divides countries and regions around the world into four categories according to the human development index (HDI): very high, high, medium and low HDI. Most ovarian cancer patients live in very high and high HDI regions. The incidence of ovarian cancer is increasing with the increase in HDI (2).

At present, effective means of screening for ovarian cancer at an early stage are still lacking; this disease at the beginning stage is not visible and the first symptoms are not evident. Approximately 75% of patients are already in the advanced stage at the moment of the diagnosis (3), and the 5-year survival rate is only 39%, while the 5-year survival rate of patients at an early stage of this tumor reaches 71% to 93% (4). Some scholars proposed that the sensitivity and specificity of ovarian cancer diagnosis can be improved by the combined monitoring of other indicators. At present, a variety of assessment models have been developed based on serum CA125 and (or) HE4 levels combined with indicators including patient’s menopausal status to predict the risk of ovarian cancer in patients with suspected benign ovarian tumors, showing good diagnostic value (5). The survival benefit of ovarian cancer patients has been significantly improved, but the prognosis is still unsatisfactory. Generally, the International Federation of Gynecology and Obstetrics (FIGO) staging (6), tumor cell reduction surgery (7), the relationship among preoperative CA125 levels, FIGO staging, survival (8), platelet count (9), and sensitivity to chemotherapeutic drugs are the basic factors in the prediction of the prognosis of ovarian cancer patients. However, the survival rate and treatment response of patients with similar clinical characteristics vary greatly due to the highly heterogeneous characteristics of this tumor with complex molecular features and genetic material changes; thus, it is particularly important to perform a prognostic assessment through the investigation of the molecular features.

Genomic instability, mutation, and metabolic reprogramming are hallmarks that influence tumor growth (10). Mitochondria are semi-autonomous organelles composed of proteins encoded by both the mitochondrial genome (mtDNA) and the nuclear genome. 99% of the proteins are encoded by the nuclear genome, while the remaining 1% is encoded by mtDNA. In principle, mutations in mtDNA or nuclear-encoded mitochondrial genes cause mitochondrial dysfunction, leading to the occurrence and development of tumors. Otto Warburg (11) was the first reporting a metabolic phenomenon different from that of normal cells: tumor cells still rely on glycolysis to produce large amounts of lactic acid under aerobic conditions. This phenomenon is called the “Warburg effect” due to mitochondrial dysfunction caused by mtDNA mutations, mitochondrial enzyme defects, or nuclear gene mutations (12).

The spread of transcriptome sequencing technology has generated a large amount of transcriptome data, which are available in public databases, providing a basis for scholars to perform prognostic research on cancers including ovarian cancer. Previous studies on the prognosis of ovarian cancer mainly focused on the nuclear genome or a single gene, while studies on the expression of mitochondrial genes and ovarian prognosis are few and incomplete. Since mitochondrial dysfunction is closely related to the occurrence and development of many tumors including ovarian cancer, it is necessary to discover mitochondrial markers for ovarian cancer prognosis and explore their clinical application value.

## Materials and methods

### Data acquisition

The TCGA-OV dataset was downloaded from the UCSC Xena database (https://gdc.xenahubs.net/), which includes 379 transcriptome original expression count data, 758 clinical information, and 731 survival information. A total of 378 ovarian cancer samples were finally included in the training set after merging and matching the above data.

The Ovarian Cancer Australia (OV-AU) dataset includes clinical information and transcriptome raw count data of 93 ovarian cancer patients; it was downloaded from the ICGC database (https://dcc.icgc.org/) and included in the validation set.

The “count2tpm” function in the “IOBR” package (13) was used to convert the raw count data of the two datasets into transcripts per kilobase million (TPM) data, and the log2-transformed [log2^(TPM+1)^] data were used for subsequent analysis.

A total of 1650 mitochondrial genes were obtained from the Mito Miner v4.0 database (http://mitominer.mrc-mbu.cam.ac.uk/).

### Screening of differentially expressed mitochondrial genes

Data of healthy ovarian tissue downloaded from the GTEx database were included since no data on healthy ovarian tissues adjacent to ovarian cancer are available in the TCGA database. The Gene Expression Profiling Interactive Analysis (GEPIA) database (14) was used to perform the analysis of DEGs on 426 TCGA ovarian cancer tissues and 88 GTEx healthy tissues to improve the accuracy of DEG analysis. The screening criteria to obtain the DEGs were fold change (FC) > 2.828 or < 0.353, i.e., |log2FC| > 1.5 and corrected *P* < 0.01. The “ggplot” function in the R “ggplot2” package was used to draw a volcano plot to visualize the related DEGs, while the “Venn” package was used to visualize the intersection of DEGs and 1,650 mitochondrial genes, and the mitochondrial DEGs were obtained.

### Screening of prognostic signature genes

The R “rms” and “survival” packages were used to perform the univariate Cox regression analysis on the DEGs related to mitochondria in the training set, and the genes related to the prognosis of ovarian cancer patients were found. The “glmnet” package was used to perform the least absolute shrinkage and selection operator (LASSO) regression analysis on the prognostic genes, and the calculated minimum λ value was used as the optimal reference value to reduce their number. Subsequently, multivariate Cox proportional hazard regression analysis was performed, and the optimal model was determined according to the minimum Akaike information criterion (AIC) value. Finally, the expression of prognostic genes in ovarian cancer tissues and healthy tissues was analyzed online using the GEPIA database.

### Clinical sample collection

Ovarian cancer and normal tissue samples were collected during 2022-2024 from 16 patients awaiting cancer surgery at the First Affiliated Hospital of the Air Force Medical University. The patient demographic and clinical information is shown in the **Supplementary Table 1**. Specimen information after tissue collection was kept strictly confidential. No patients with OV was subjected to anticancer treatment such as chemotherapy or radiotherapy prior to surgery. All tissue specimens were collected within 30 minutes of surgery, placed in liquid nitrogen, and stored at -80 ˚C. The procedures were approved by the Ethics Committee of the First Affiliated Hospital of the Air Force Medical University and signed informed consent was obtained from each patient prior to the collection of tissue samples.

### Quantitative real-time PCR

Total RNA was extracted from 8 ovarian cancer tissues, including 2 grade II, 3 grade III, and 3 grade IV, and 8 paired normal tissues using TRIzol Takara, Kyoto, Japan)according to the manufacturer’s instructions. Complementary DNA (cDNA) was synthesized using PrimeScript TM RT reagent kit with gDNA Eraser kit (RR047A, Takara). Then, the product was quantified using SYBR Premium Ex Taq II and ABI Life technologies. GAPDH was used as the housekeeping gene for the calculation of relative gene expression. The primers are listed in the **Supplementary Table 2**.

### Hematoxylin-eosin and Sirius red staining

The collected healthy and cancer ovarian tissues were fixed in a 4% paraformaldehyde solution for 4 hours and dehydrated overnight with 30% sucrose. OTC embedding, liquid nitrogen freezing and storage at -80°C was performed prior to frozen sectioning into 5 µm-thick sections on a cryostat. Hematoxylin-eosin staining was performed on the Nor group and OV group.

### Immunofluorescence

The changes in the proportion of M1 type (CD11b+ CD86+) and M2 type macrophages (CD11b+CD206+) were evaluated in normal ovarian tissue and ovarian cancer tissue by dual immunofluorescence. The 5 µm-thick frozen sections were fixed in methanol and blocked by goat serum. Next, the sections were treated with the following primary recombinant monoclonal antibodies: rabbit anti-CD11b antibody (Abcam, ab133357, 1:300 dilution), mouse anti-CD86 (PTM BIO, PTM-5334, 1:100 dilution), and mouse anti-CD206 (SANTA CRUZ, sc-58986, 1:100 dilution). Then, they were treated with the secondary antibodies CoraLite488-conjugated Goat Anti Rabbit IgG (Protentech, SA00013-2) and CoraLite594-conjugated Goat Anti Mouse IgG (Protentech, SA00013-3). At least 5 different regions were selected for image acquisition for each sample and positive cells were quantified using Image Pro Plus 6.0 software.

### Construction and validation of the prognostic risk model

The regression coefficient of the prognostic feature gene was determined using the “Coxph” function in the R “survival” package, and the linear combination method of the gene expression multiplied by the regression coefficient was used to construct the prognostic risk model. The specific risk score formula was as follows:


(1) $$\text{Risk Score}=\sum_{\text{i}=1}^{\text{n}}\left({\text{β}}_{\text{i}}\times {\text{Exp}}_{\text{i}}\right)$$

in which β is the regression coefficient, Exp is the expression of the characteristic genes and i is the number of characteristic genes.

The introduction of the screened gene expression values into Equation 1 led to the risk score of each ovarian cancer patient. The median of the risk score was used as the cutoff value to classify the ovarian cancer patients into the high-risk group and the low-risk group in the training and validation set. The “surv_cutpoint” function of the “survminer” package was used to determine the optimal cutoff value of the expression of the above-mentioned prognostic genes, and the patients were divided into high-expression group and low-expression group according to their respective cutoff values.

The KM curve was plotted using the “ggsurvplot” function in the R “survival” package to assess the survival status of the two groups. Subsequently, the R “timeROC” package was used to plot the ROC curve to evaluate the accuracy of the model prediction. Finally, the “ggplot2” package in R was used to plot the risk score association diagram to visualize the risk score, survival time, and survival status of each patient.

### Construction and evaluation of composite nomograms

The univariate Cox regression analysis was used to analyze the predictors significantly related to prognosis, such as age, FIGO stage, residual tumor status, vascular invasion, lymph node invasion, tumor grade and other clinical factors and risk scores (*P* < 0.05). Then, multivariate Cox proportional hazards regression analysis was performed to identify predictors significantly associated with prognosis (*P* < 0.05), and a composite nomogram was constructed based on the R “rms” package. A total score was obtained for each patient by adding the scores of the predictors in the nomogram, and the patient’s survival outcome at 1, 3, and 5 years was predicted. In addition, ROC curve, calibration curve and decision curve analysis were used to evaluate the predictability, accuracy and clinical utility of the nomogram.

### Differential gene function enrichment analysis

The function and effect of mitochondrial DEGs were analyzed using Gene Ontology, GO and Kyoto Encyclopedia of Genes and Genomes (KEGG) databases and the R “clusterProfiler”, “org.Hs.eg.db”, “enrichplot” and “ggplot2” packages.

### Mutation analysis

A total of 436 somatic mutation data (MuTect2 Variant Aggregation and Masking) were downloaded from the UCSC Xena database TCGA-OV data set, and patients with incomplete clinical and survival information were removed. Finally, mutation analysis was performed using the included mutation data of 271 ovarian cancer patients. Mutation waterfall plots of the high-risk and low-risk groups were plotted using the R “maftools” package, and the tumor mutation burden (TMB) score of each patient was calculated. The 271 patients were divided into a high TMB group and low TMB group according to the median TMB score.

### Analysis of immune infiltration and immune efficacy response

A total of 591 immunophenotype score (IPS) data of ovarian cancer patients were downloaded from the TCIA database using the Cancer Immunome Atlas. They were then merged with the data of the 378 patients in this study. Finally, 153 patients were included for immune analysis, including cases in the high-risk group and 70 in the low-risk group.

The Tumor Immune Dysfunction and Exclusion (TIDE) database predicts patients’ responses to immune checkpoint inhibitors such as anti-PD-1 and anti-CTLA-4 by assessing the possibility of tumor immune escape (15). The lower the TIDE score, the lower the possibility of tumor immune escape and the better the efficacy of immune checkpoint inhibitors.

IMvigor210 is a single-arm phase II clinical trial for the evaluation of the efficacy and safety of PD-L1 inhibitors in patients with urothelial carcinoma (16). The complete immunotherapy clinical data of 298 cases are stored in the IMvigor210 CoreBiologies package (http://research-pub.gene.com/IMvigor 210CoreBiologies/). Immunotherapy response included complete response (CR), partial response (PR), stable disease (SD) and progressive disease (PD). Patients who achieved CR or PR were classified as responders, while those who achieved SD or PD were classified as non-responders. The expressions of the genes determined by the model in the IMvigor210 cohort were placed into the Equation 1 to obtain the patient’s risk score. Next, the patients were divided into a high-risk group and low-risk group based on the median risk score.

The TCIA database, TIDE database and IMvigor210 dataset were used to explore the relationship between the mitochondrial risk score and the immunotherapy response.

### Drug sensitivity analysis

The Genomics of Drug Sensitivity in Cancer (GDSC) database was used to perform drug sensitivity analysis. The GDSC v2 dataset was obtained using the R “OncoPredict” package, and a ridge regression model was constructed using the “calcPhenotype” function. This model predicted the drug sensitivity of 378 patients in the training set and the half maximal inhibitory concentration (IC50) data of 198 drugs were obtained (17). The ability of the risk score to predict the response to 198 chemotherapeutic drugs was explored by comparing their IC50 between the two risk groups.

### Statistical analysis

GraphPad Prism 8.0.2 and R 4.3.1 software were used for data entry and processing. Data with normal distribution in two groups were compared using Student *t*-test and the results were expressed as mean ± standard deviation. Data not normally distributed were compared using the Mann-Whitney U test between two groups of continuous variables and the results were expressed as the median (quartile). Categorical variables were expressed as frequencies (percentages), and differences between groups were assessed using 2’s test or Fisher’s exact test. Spearman rank correlation coefficient was used to evaluate the potential presence of a significant correlation between the two groups of variables. Survival curves were plotted according to the KM method, and survival rates were compared using the log-rank test. Cox proportional hazards regression model was used for univariate and multivariate analysis, and the results were expressed as hazard ratios (HR) and 95% confidence intervals (95% CI). A value of *P* < 0.05 was considered statistically significant.*P<0.05, **P<0.01, ***p<0.001.

## Results

### Identification and functional enrichment analysis of mitochondrial DEGs in ovarian cancer tissues

**Figure 1:**
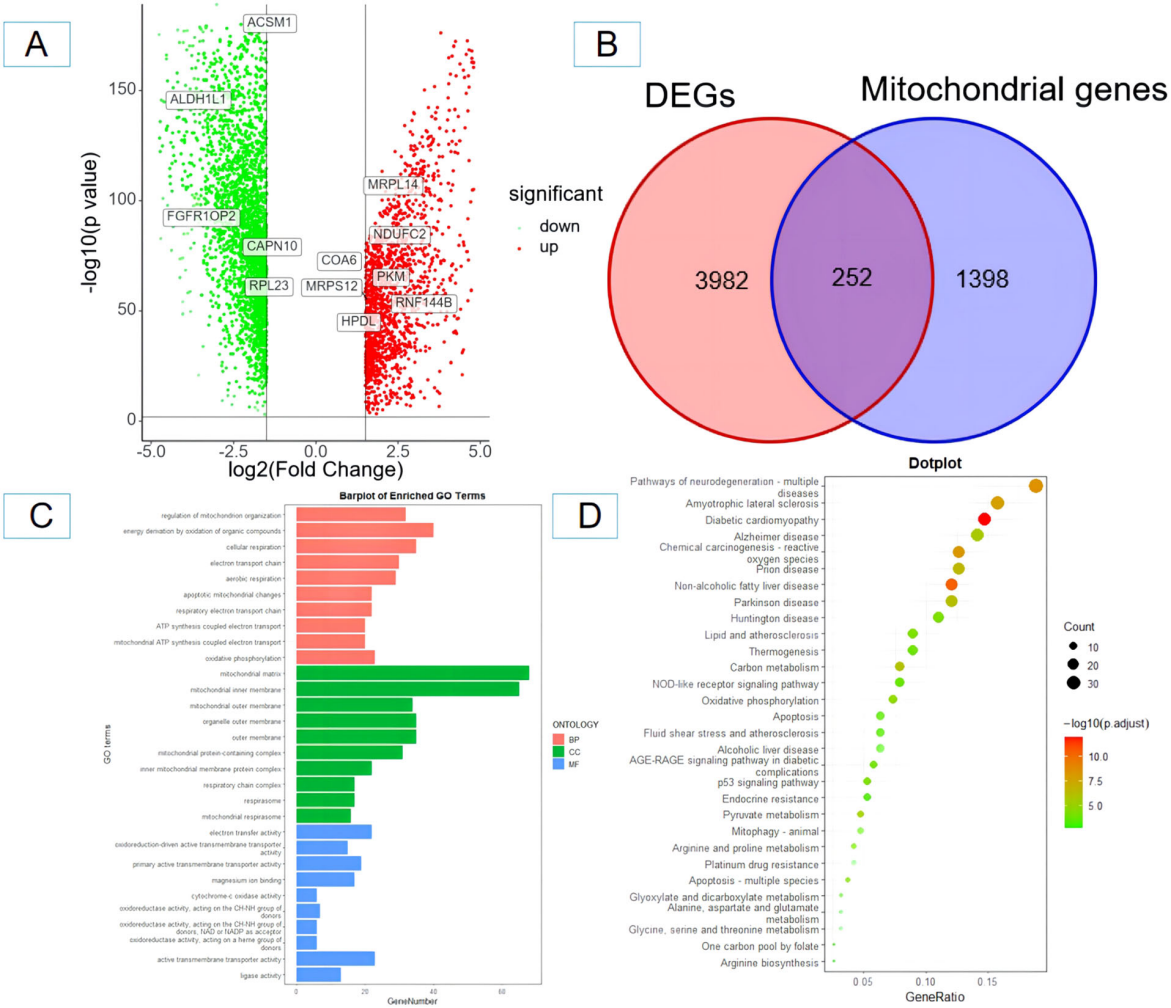
Screening and identification of mitochondrial-related differentially expressed genes in ovarian cancer tissues and enrichment analysis. **(A)** Volcano plot of differentially expressed genes (red and yellow points in the figure indicate significantly up-regulated and significantly down-regulated differentially expressed genes); **(B)** Venn diagram of differentially expressed genes and mitochondrial-related genes. **(C)** GO functional enrichment analysis of mitochondrial-related differentially expressed genes in ovarian cancer; **(D)** KEGG pathway enrichment analysis of mitochondrial-related differentially expressed genes in ovarian cancer. BP, biological process; CC, cellular component; MF, molecular function.

A total of 4,234 DEGs between ovarian cancer tissues and healthy tissues were found using the GEPIA database; 1,501 were upregulated and 2,733 were downregulated (**Figure 1A**). Subsequently, the 4,234 DEGs were intersected with the 1,650 mitochondrial genes, and finally, 252 mitochondrial DEGs were obtained (**Figure 1B**). The biological significance of mitochondrial DEGs was established by performing the GO function and KEGG pathway enrichment analysis. The former showed that 252 mitochondrial DEGs were related to several biological processes including the regulation of mitochondrial organization, cellular respiration, and electron transport chain. In addition, they were mainly related to cellular components including mitochondrial matrix, mitochondrial outer membrane, and mitochondrial inner membrane. Finally, they were related to several molecular functions including transmembrane transporter activity, oxidoreductase activity and lipase activity (**Figure 1C**). The KEGG channel analysis showed that the 252 mitochondrial DEGs were mainly involved in important pathways such as neurodegeneration, p53 signaling pathway, NOD-like receptor signaling pathway, lipid and atherosclerosis, reactive oxygen species, oxidative phosphorylation metabolism, and apoptosis (**Figure 1D**).

### Construction and validation of the prognostic risk model for ovarian cancer

**Figure 2:**
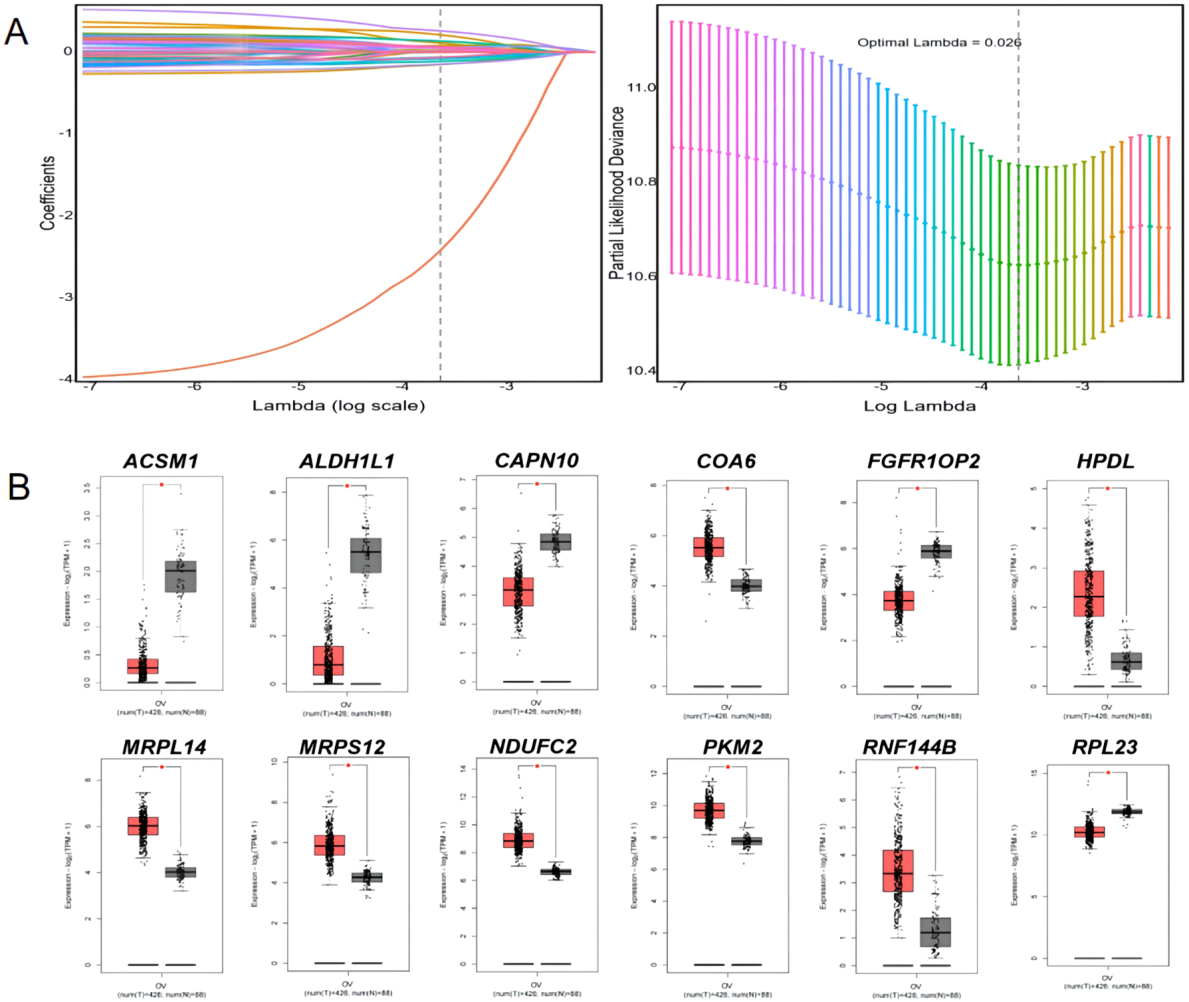
LASSO regression analysis and expression of 12 prognostic signature genes in ovarian cancer tissues and normal tissues. **(A)** LASSO regression analysis; **(B)** and expression of 12 prognostic signature genes in ovarian cancer tissues and normal tissues. * p<0.05.

**Figure 3:**
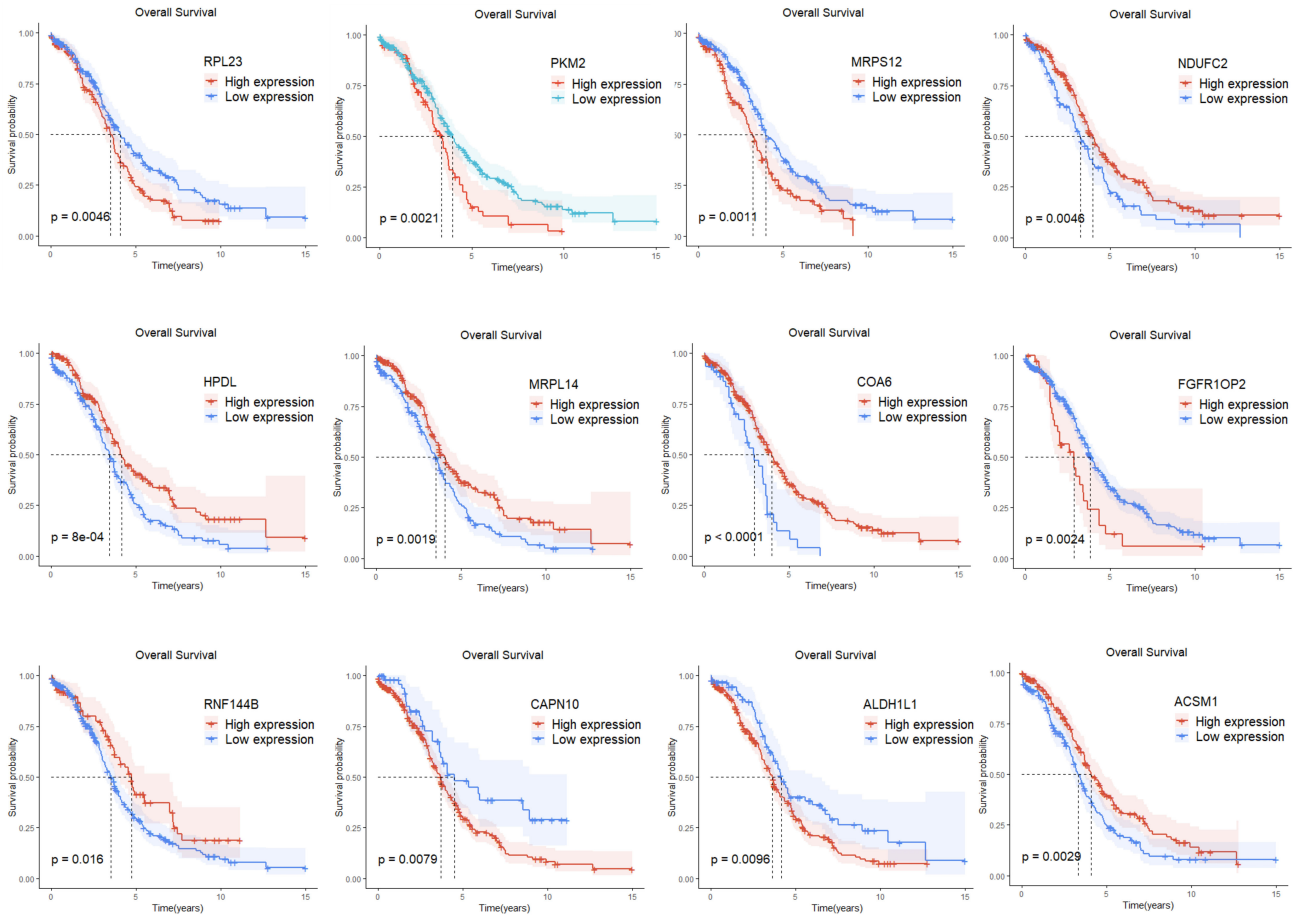
KM analysis of 12 prognostic signature genes.

Single-factor Cox regression analysis performed on the 252 mitochondrial differential genes in the training set revealed that a total of 42 genes were significantly related to prognosis (**Table 1**). Subsequently, prognostic genes were further screened by LASSO regression analysis, α=1 was selected, and the ten-fold cross-validation method was used to achieve the internal validation of the model. Fourteen prognostic genes were screened when λ min was 0.026 (**Figure 2A**). Finally, 12 characteristic genes related to the prognosis of ovarian cancer were identified based on the minimum AIC value (2262.89), such as RPL23, PKM2, MRPS12, NDUFC2, HPDL, MRPL14, COA6, FGFR1OP2, RNF144B, CAPN10, ALDH1L1, and ACSM1. PKM2, MRPS12, NDUFC2, HPDL, MRPL14, COA6 and RNF144B were significantly upregulated in ovarian cancer tissues than in normal tissues (*P* < 0.05, **Figure 2B**), while RPL23, FGFR1OP2, CAPN10, ALDH1L1 and ACSM1 were significantly down-regulated (*P* < 0.05). In addition, the KM analysis showed that the OS of patients with high expression of RPL23 (p=0.005), PKM2 (p=0.002), MRPS12 (p= 0.001), FGFR1OP2 (p=0.002), CAPN10 (p=0.008) and ALDH1L1 (p=0.01) The OS of NDUFC2 (p=0.005), HPDL (p=0.001), MRPL14 (p=0.002), COA6(*P*< 0.0001), RNF144B (p=0.016) and ACSM1 (p=0.003) was significantly longer than that of patients with low expression (**Figure 3**). Finally, multifactor Cox regression analysis was performed on the 12 prognostic genes (**Table 2**), and the obtained regression coefficients were placed into the Equation 1 to construct the risk score model.

**Table 1:** Results of univariate Cox regression analysis.

Gene	*HR*	95% *CI*		*P*-value
		Lower	Upper	
*RPL3*	1.2	1.0	1.45	0.056
*IFI6*	0.93	0.87	1.0	0.065
*RPL23*	1.27	1.06	1.52	0.009
*ENO1*	1.17	0.98	1.39	0.080
*PKM2*	1.21	1.0	1.47	0.047
*IFI27*	0.94	0.88	1.0	0.049
*DDIT4*	1.14	1.03	1.26	0.014
*MRPS12*	1.18	1.0	1.37	0.043
*UQCRFS1*	1.19	1.01	1.41	0.037
*PPP1R15A*	1.25	1.08	1.44	0.003
*NDUFC2*	0.82	0.69	0.98	0.026
*HPDL*	0.89	0.79	1.0	0.042
*MRPL14*	0.78	0.63	0.96	0.018
*RSAD1*	1.23	1.02	1.49	0.027
*DMPK*	1.18	1.0	1.38	0.045
*HARS2*	1.19	0.97	1.46	0.091
*OCIAD2*	0.85	0.71	1.02	0.074
*COA6*	0.79	0.63	0.98	0.035
*TIMM23*	0.77	0.58	1.01	0.061
*PDK4*	1.19	1.03	1.36	0.016
*WDR81*	1.2	1.04	1.38	0.013
*MTX3*	1.28	1.05	1.57	0.015
*OGDHL*	1.15	1.01	1.3	0.036
*LSS*	1.17	0.99	1.39	0.071
*FGFR1OP2*	1.27	1.03	1.55	0.022
*D2HGDH*	1.28	1.08	1.53	0.006
*RNF144B*	0.85	0.74	0.98	0.029
*FBXO17*	1.14	1.01	1.28	0.031
*SLC25A37*	1.21	0.99	1.49	0.06
*ACSS1*	1.13	0.98	1.31	0.099
*CAPN10*	1.31	1.03	1.66	0.028
*SLC25A25*	1.17	0.98	1.38	0.075
*SLC25A42*	1.24	0.97	1.58	0.085
*PAPSS2*	1.26	0.96	1.66	0.097
*SH3BP5*	1.37	1.03	1.84	0.034
*SERHL2*	1.45	1.05	2.02	0.024
*ACACB*	1.51	1.08	2.09	0.015
*METAP1D*	1.45	0.95	2.2	0.083
*MSRB3*	1.41	0.98	2.01	0.062
*ACSS3*	1.61	1.03	2.53	0.038
*ALDH1L1*	1.62	1.1	2.38	0.014
*PDE2A*	1.53	0.94	2.49	0.086
*ACSM1*	0.18	0.03	1.06	0.058

**Table 2:** Results of multivariate Cox regression analysis.

Gene	*β*	*HR*	95% *CI*	*P*-value
*RPL23*	0.348	1.42	1.18∼1.71	0.0003
*PKM2*	0.247	1.28	1.03∼1.6	0.0281
*MRPS12*	0.203	1.23	1.03∼1.45	0.0183
*NDUFC2*	−0.184	0.83	0.69∼1	0.0468
*HPDL*	−0.129	0.88	0.77∼1	0.0517
*MRPL14*	−0.219	0.8	0.61∼1.06	0.1232
*COA6*	−0.272	0.76	0.58∼1	0.0541
*FGFR1OP2*	0.253	1.29	1.05∼1.59	0.0173
*RNF144B*	−0.217	0.81	0.69∼0.94	0.0058
*CAPN10*	0.370	1.45	1.08∼1.95	0.0145
*ALDH1L1*	0.342	1.41	0.92∼2.15	0.1148
*ACSM1*	−3.780	0.02	0∼0.18	0.0003


$$\begin{array}{l} \text{Risk Score}=\left(0.348\right)\times \text{Exp}\left(\text{RPL}23\right)+\left(0.247\right)\times \text{Exp}\left(\text{PKM}2\right)+\left(0.203\right)\times \text{Exp}\left(\text{MRPS}12\right)\\ +\left(-0.184\right)\times \text{Exp}\left(\text{NDUFC}2\right)+\left(-0.129\right)\times \text{Exp}\left(\text{HPDL}\right)+\left(-0.219\right)\times \text{Exp}\left(\text{MRPL}14\right)\\ +\left(-0.272\right)\times \text{Exp}\left(\text{COA}6\right)+\left(0.253\right)\times \text{Exp}\left(\text{FGFR}1\text{OP}2\right)+\left(-0.217\right)\times \text{Exp}\left(\text{RNF}144\text{B}\right)\\ +\left(0.370\right)\times \text{Exp}\left(\text{CAPN}10\right)+\left(0.342\right)\times \text{Exp}\left(\text{ALDH}1\text{L}1\right)+\left(-3.780\right)\times \text{Exp}\left(\text{ACSM}1\right) \end{array}$$

### Q-OPCR validation of 12 prognostic signature genes

**Figure 4:**
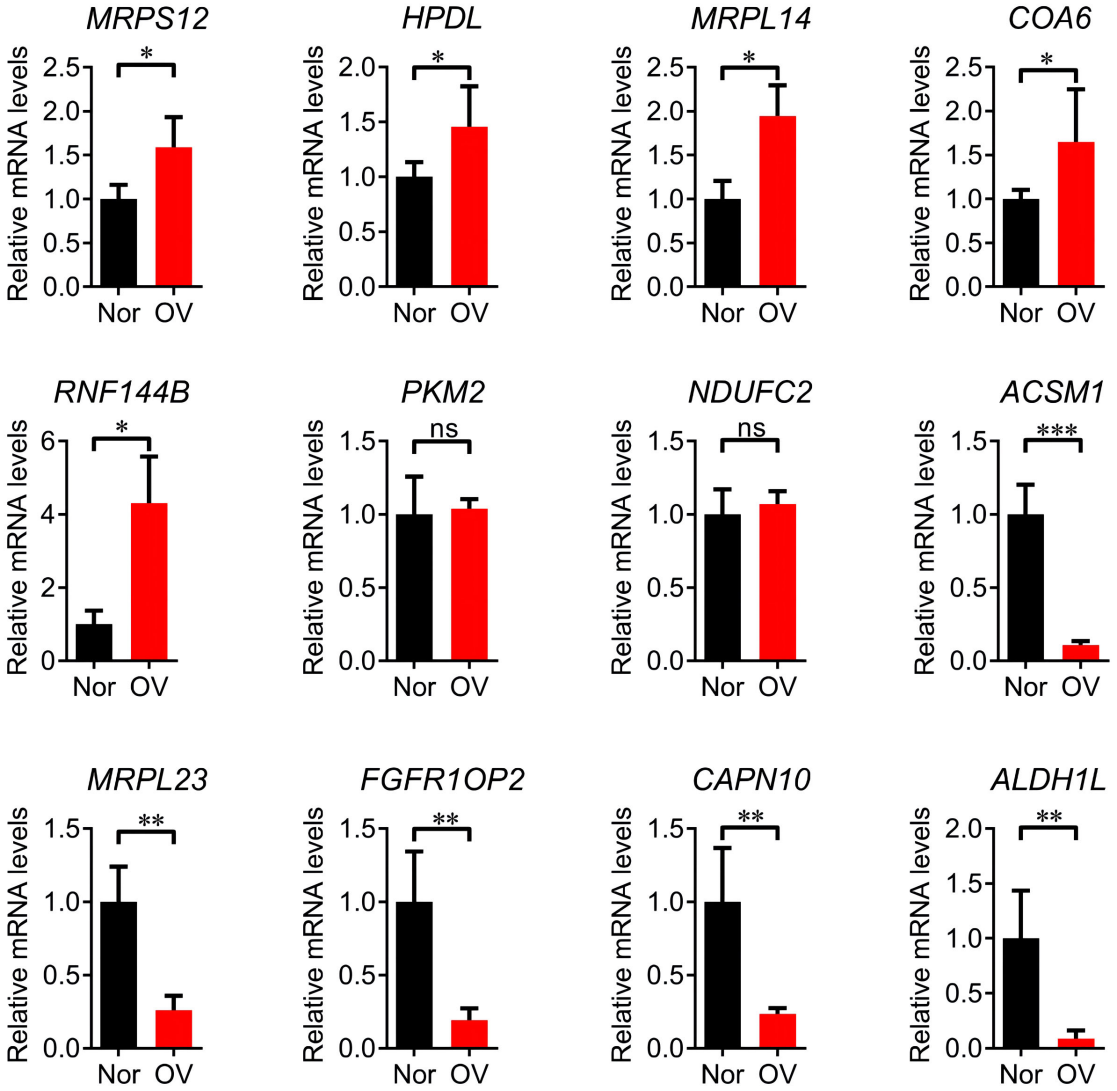
Q-PCR validation analysis of 12 prognostic signature genes. * p<0.05, ** p<0.01, *** p<0.001, ns p>0.05.

Compared with normal tissues, MRPS12, NDUFC2, HPDL, MRPL14, COA6 and RNF144B were significantly up-regulated in ovarian cancer tissues (P<0.05), RPL23, FGFR1OP2, CAPN10, ALDH1L1 and ACSM1 were significantly down-regulated (P<0.05) and the up-regulation of PKM2 and NDUFC2 was no significant (**Figure 4**).The treatment status of the sample of 8 patients is shown in **Supplementary Table 3**.

**Figure 5:**
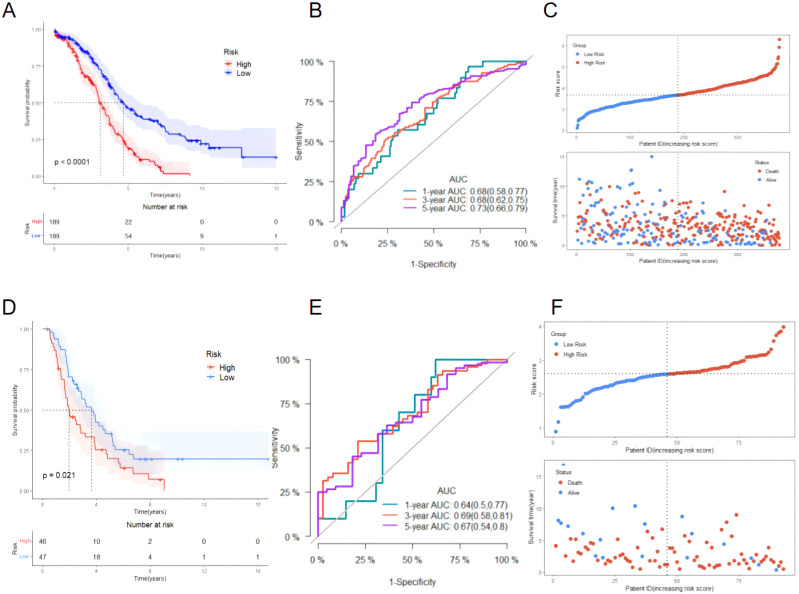
Evaluating the performance of the prognostic risk model in the training set and evaluating the performance of the prognostic risk model in the validation set. **(A)** KM survival curve distribution of the prognostic risk model in the training set; **(B)** ROC curve of the prognostic risk model in the training set; **(C)** Risk factor linkage diagram (top, risk score scatter plot; bottom, patient life and death scatter plot). **(D)** KM survival curve distribution of the prognostic risk model in the validation set; **(E)** ROC curve of the prognostic risk model in the validation set; **(F)** Risk factor linkage diagram (top, risk score scatter plot; bottom, patient life and death scatter plot).

According to the risk score obtained from the Equation 1, the training set was divided into a high-risk group (189 cases) and a low-risk group (189 cases) using the median risk score 3.67 as the cutoff value. The KM survival analysis showed that the OS of patients in the high-risk group was significantly shorter than that of patients in the low-risk group (*P* < 0.0001, **Figure 5A**). The accuracy of the prognostic risk model in predicting 1, 3, and 5-year OS was further evaluated by plotting the ROC curve. The AUC values at 1, 3, and 5 years were 0.68, 0.68, and 0.73, respectively (**Figure 5B**). The number of ovarian cancer patients who died in the high-risk group was higher than that in the low-risk group, while the number of ovarian cancer patients who survived for more than 5 years in the low-risk group was higher than that in the high-risk group (**Figure 5C**). The robustness of the prognostic risk model was assessed by evaluating the predictive value of 12 mitochondrial genes in the validation set. The risk score of each ovarian cancer patient in the validation set was obtained by inserting the expression of 12 genes into the Equation 1, and using the median risk score 2.59 as the cutoff value; in this way, the validation set was divided into high-risk group (46 cases) and low-risk group (47 cases). The researches show that consistent with the results of the training set, the OS of patients in the high-risk group was significantly shorter than that of patients in the low-risk group (*P* = 0.021, **Figure 5D**). ROC curve analysis showed that the AUC values in 1, 3, and 5 years were 0.64, 0.69, and 0.67, respectively (**Figure 5E**). The patient’s prognosis becomes worse as the patient’s risk score increased (**Figure 5F**). These results indicated a good performance of the prognostic risk model constructed based on 12 mitochondrial genes.

The comparison of the clinicopathological characteristics between high-risk and low-risk groups showed that the death rate of patients in the high-risk group (70.37% *vs*. 52.38%) was significantly higher than that in the low-risk group (*P* < 0.05). However, there was no statistically significant difference in age, FIGO stage, tumor residual status, vascular invasion, lymph node invasion, and tissue grade between the two groups (**Table 3**).

**Table 3:** Correlation between risk scores and clinical pathological characteristics of patients in the training set.

Variables	Risk Score		*P*-value
	Low, *n* (%) *n*=189	High, *n* (%) *n*=189	
Age (year)			0.410
<60	103 (54.50)	95 (50.26)	
≥60	86 (45.50)	94 (49.74)	
FIGO stage			0.421
I/I	13 (6.88)	11 (5.82)	
III/IV	176 (93.12)	178 (94.18)	
Residual tumor			0.167
R0	49 (25.93)	35 (18.52)	
R1	120 (63.49)	127 (67.20)	
N/A	20 (10.58)	27 (14.29)	
Venous invasion			0.389
Yes	37 (19.58)	27 (14.29)	
No	20 (10.58)	21 (11.11)	
N/A	132 (69.84)	141 (74.60)	
Lymphatic invasion			0.952
Yes	51 (26.98)	50 (26.46)	
No	23 (12.17)	25 (13.23)	
N/A	115 (60.85)	114 (60.32)	
Grade			0.386
G2	25 (13.23)	20 (10.58)	
G3	158 (83.60)	163 (86.24)	
G4	0 (0.00)	2 (1.06)	
GX	6 (3.17)	4 (2.12)	
Outcome			<0.001
Survival	90 (47.62)	56 (29.63)	
Non-survival	99 (52.38)	133 (70.37)	

Bold values indicate significant difference with p<0.05, and a highly significant difference with p<0.01.

### Construction and evaluation of composite nomograms

**Figure 6:**
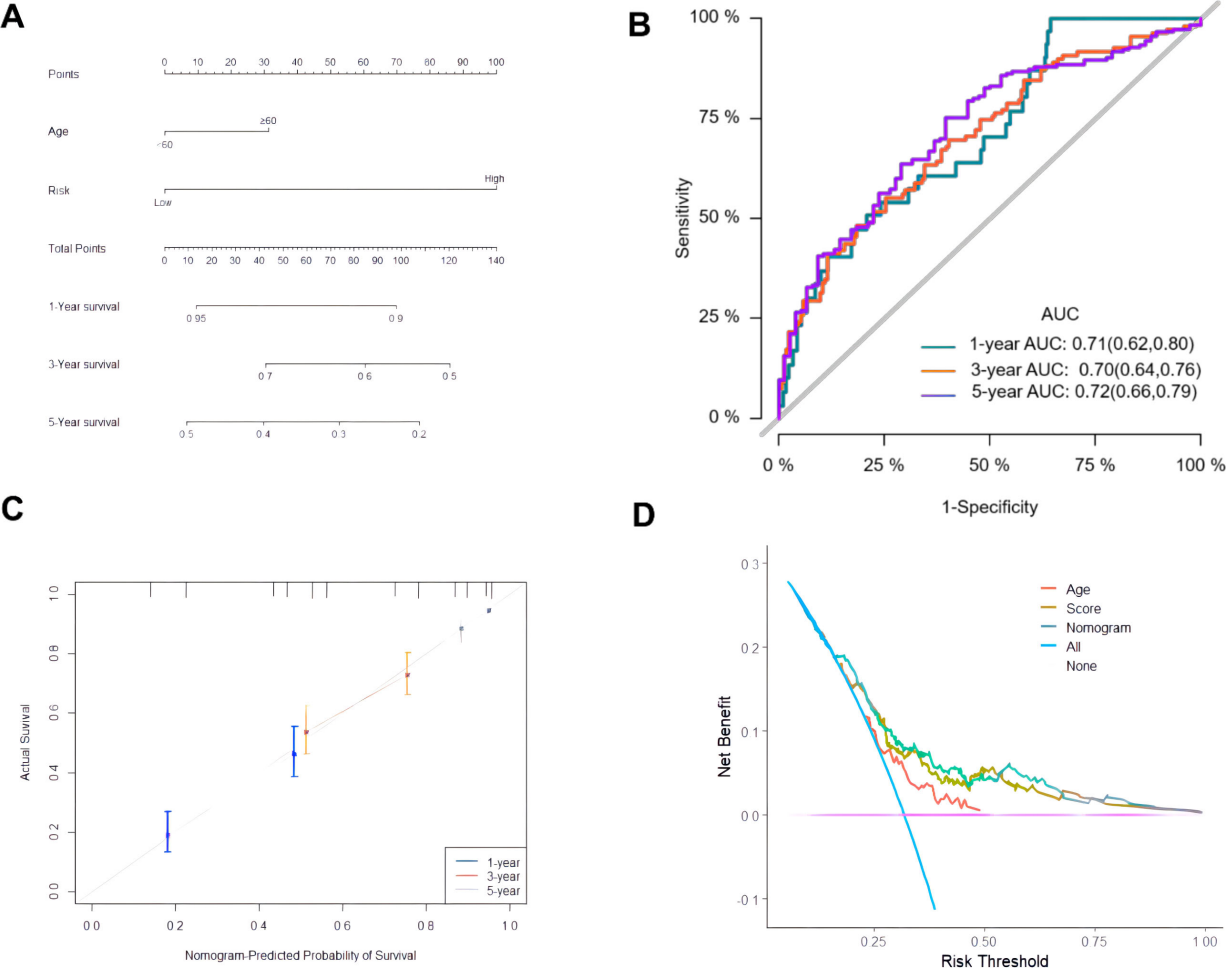
Construction and evaluation of composite nomogram. **(A)** Construction of a composite nomogram based on age and risk score; **(B)** ROC curve of the composite nomogram model; **(C)** Calibration curve; **(D)** DCA curve.

Univariate and multivariate Cox analysis was performed on 378 ovarian cancer patients to evaluate whether the prognostic risk model could be used as an independent prognostic factor for ovarian cancer patients. Age, tumor residual status and risk score were associated with OS in ovarian cancer patients as revealed by the univariate Cox regression analysis (**Table 4**), while age (*P* = 0.0412) and risk score (*P* < 0.001) were independent prognostic factors affecting OS in ovarian cancer patients as revealed by the multivariate Cox regression analysis (**Table 4**). A composite nomogram was constructed based on risk score and age, as shown in **Figure 6A**. The ROC curve showed that the AUC values of 1, 3 and 5 years were 0.71, 0.70 and 0.72, respectively (**Figure 6B**). The calibration curve showed that the actual survival probability of 1, 3 and 5 years was almost consistent with the survival probability predicted by the nomogram model (**Figure 6C**). The decision curve analysis showed that the nomogram model was superior to age and risk score in predicting the prognosis of ovarian cancer patients (**Figure 6D**). These results indicated that the nomogram model constructed based on risk score and age might have a strong clinical applicability and accurate predictive power.

**Table 4:** Cox regression analysis of factors affecting survival in 378 patients with ovarian cancer.

Variables	Patient (*n*=378)	Univariate analysis		Multivariate analysis	
		*HR* (95% *CI*)	*P*-value	*HR* (95% *CI*)	*P*-value
Age (years)					
<60	180	1		1	
≥60	198	1.32 (1.02~1.71)	**0.036**	1.31(1.01~1.69)	**0.0412**
FIGO stage					
I/I	24	1			
III/IV	354	2.15 (0.95~4.83)	0.066		
Residual tumor					
R0	84	1			
R1	247	8.28(4.49~15.26)	**0.001**		
N/A	47	12.51(6.21~25.23)	**0.001**		
Venous invasion					
Yes	64	1			
No	41	1.12 (0.61~2.06)	0.708		
N/A	273	1.51 (1~2.3)	0.052		
Lymphatic invasion					
Yes	101	1			
No	48	0.74 (0.44~1.26)	0.268		
N/A	229	0.92 (0.66~1.28)	0.631		
Grade					
G2	45	1			
G3	321	1.24 (0.84~1.83)	0.288		
G4	2	1.64 (0.22~12.08)	0.627		
GX	10	1.6 (0.66~3.87)	0.295		
Risk Score					
High	189	1			
Low	189	0.42(0.32~0.56)	**0.001**	0.43(0.32~0.56)	**0.001**

Bold values indicate significant difference with p<0.05, and a highly significant difference with p<0.01.

### Mutational landscape of ovarian cancer patients in the high-risk and low-risk group

**Figure 7:**
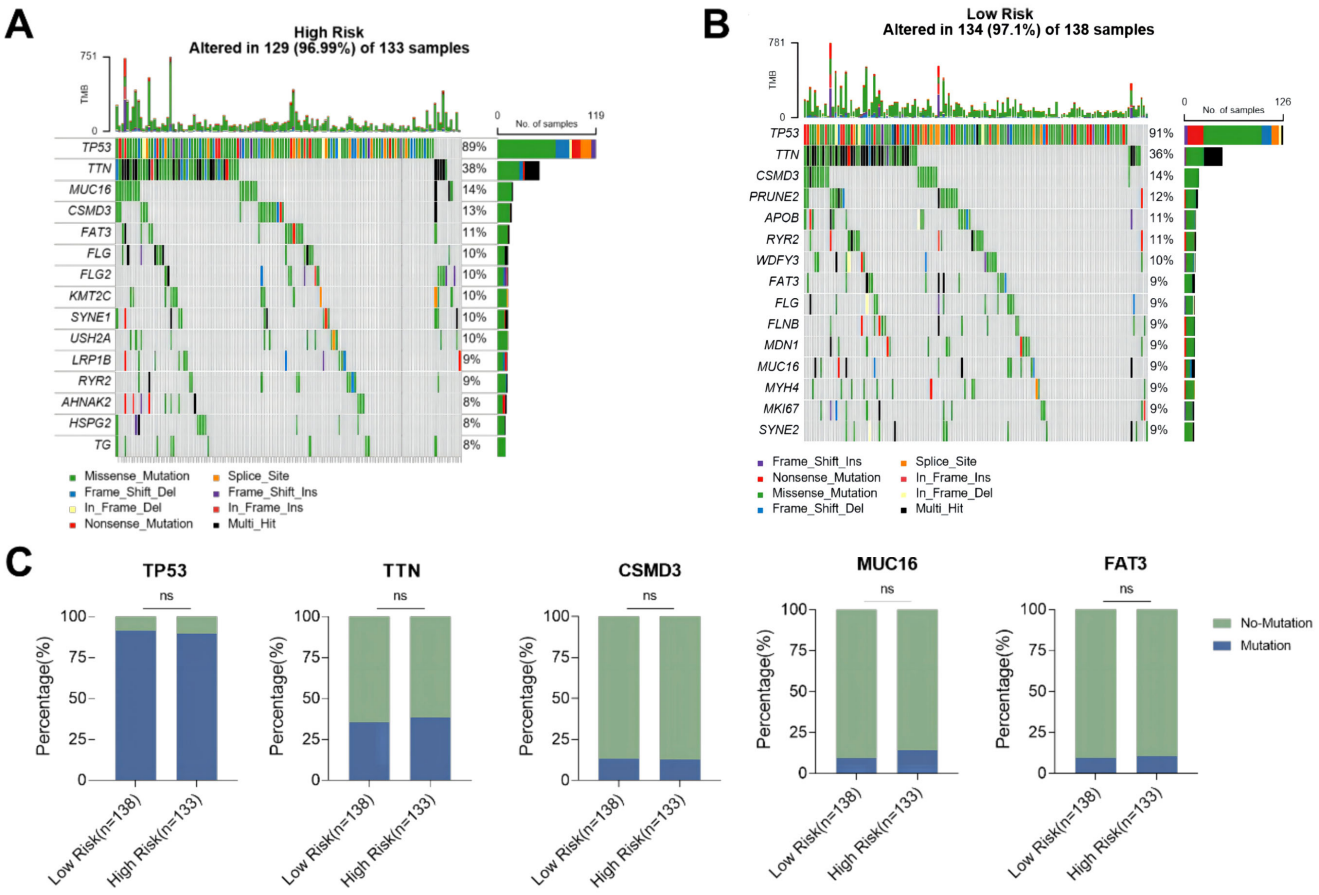
Mutation status of ovarian cancer patients in high-risk and low-risk groups. **(A)** Waterfall plot of the top 15 mutant genes in the high-risk group; **(B)** Waterfall plot of the top 15 mutant genes in the low-risk group; **(C)** Comparison of mutation rates of the top 5 mutant genes in the high-risk group and the low-risk group. ns p>0.05.

The difference in genomic mutations between the high-risk group and the low-risk group was assessed by plotting the mutation maps of ovarian cancer patients in the high-risk group (133 cases) and low-risk group (138 cases). The waterfall chart showed the top 15 mutated genes and their mutation types (**Figures 7A, B**). Among them, 7 genes including TP53, TTN, CSMD3, RYR2, MUC16, FAT3 and FLG were the most frequently mutated shared by the two groups. No significant difference was observed in the mutation rate of the top 5 mutated genes in the high-risk group compared with the low-risk group (**Figure 7C**).

**Figure 8:**
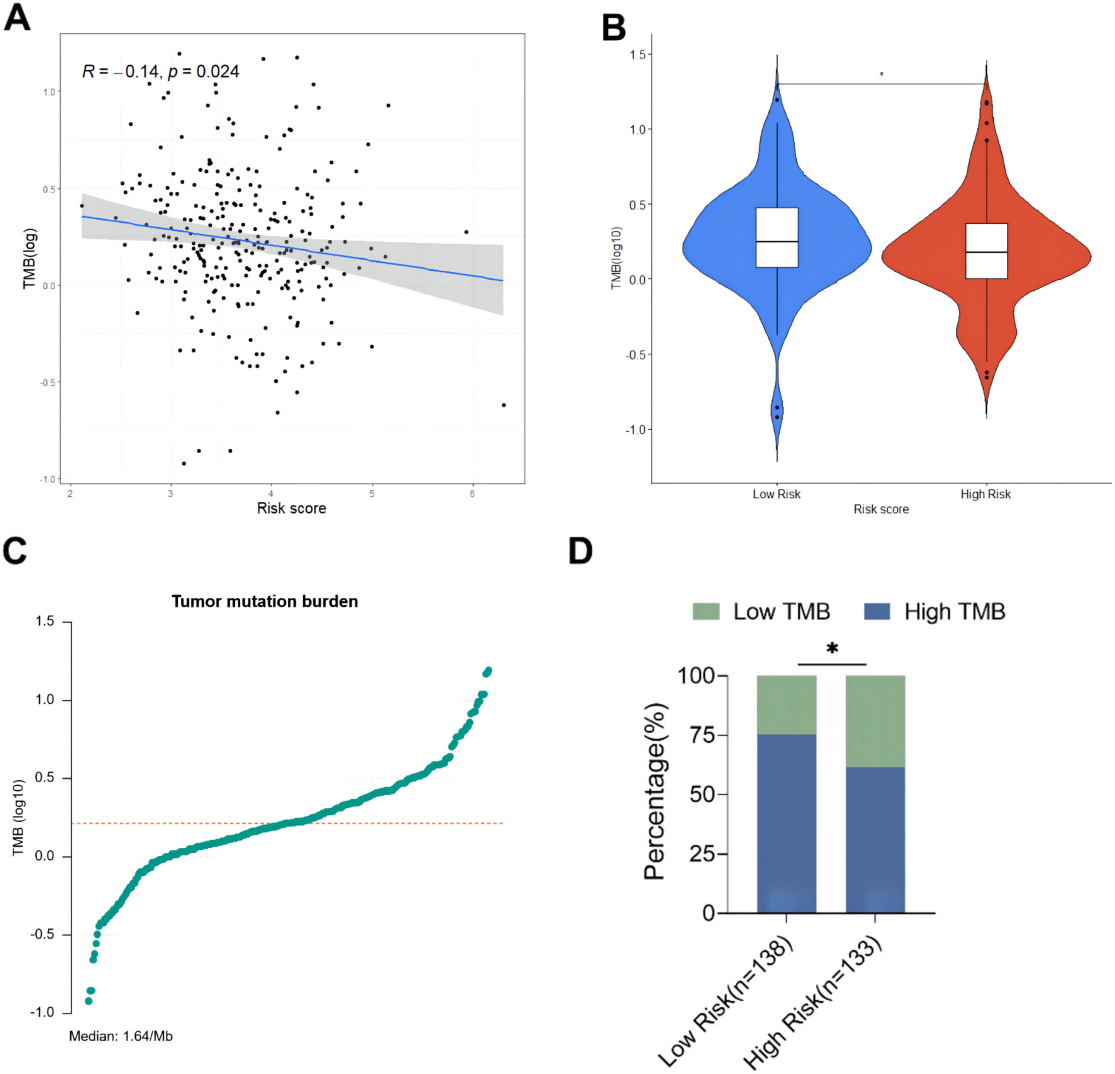
Exploring the relationship between tumor burden and risk score. **(A)** Linear correlation diagram of tumor mutation burden and risk score; **(B)** Comparison of total TMB between high-risk group and low-risk group; **(C)** Distribution diagram of tumor mutation burden; **(D)** Comparison of TMB composition between high-risk group and low-risk group. * p<0.05.

**Figure 9:**
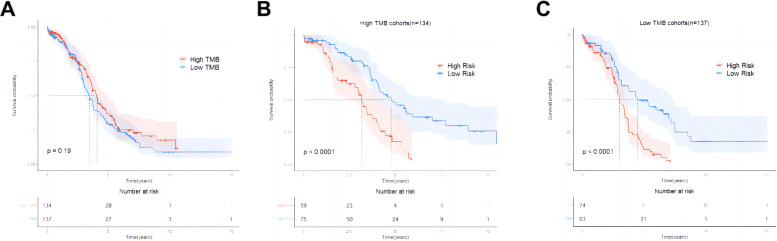
Survival curves of ovarian cancer patients in the overall population, high TMB subgroup, and low TMB subgroup. **(A)** Comparison of overall survival between the high TMB group and the low TMB group in the training set; **(B)** Comparison of overall survival between the high-risk group and the low-risk group in the subgroup of the high TMB group; **(C)** Comparison of overall survival between the high-risk group and the low-risk group in the subgroup of the low TMB group.

The relationship between risk score and TMB was further analyzed, revealing that the risk score was significantly negatively correlated with the TMB of ovarian cancer patients (**Figure 8A**). In addition, the TMB of patients in the high-risk group was significantly lower than that in the low-risk group (*P* < 0.05, **Figure 8B**). Subsequently, ovarian cancer patients were divided into high TMB group and low TMB group using the median TMB (3.64/Mb) as the cutoff value (**Figure 8C**), and the results showed a significant difference in TMB composition between the high-risk group and low-risk group (*P* < 0.05, **Figure 8D**). Tumor mutation load is a potential biomarker for immune checkpoint inhibitors in many cancer types. Therefore, this study further evaluated the correlation between TMB and prognosis of ovarian cancer patients. The comparison of the survival curves of ovarian cancer patients in the total population, high TMB subgroup and low TMB subgroup (**Figures 9A–C**) revealed that patients in the low TMB group had shorter OS than those in the high TMB subgroup, but without any statistically significant difference (*P* = 0.19). The high risk patients had significantly lower OS than the low risk patients in both the high TMB subgroup and the low TMB subgroup. These findings suggested that the combination of risk score and TMB might be a potentially valuable new marker, and that patients with a high tumor mutation load might more likely benefit from treatment.

### Correlation analysis between mitochondrial risk score and immune cell infiltration

**Figure 10:**
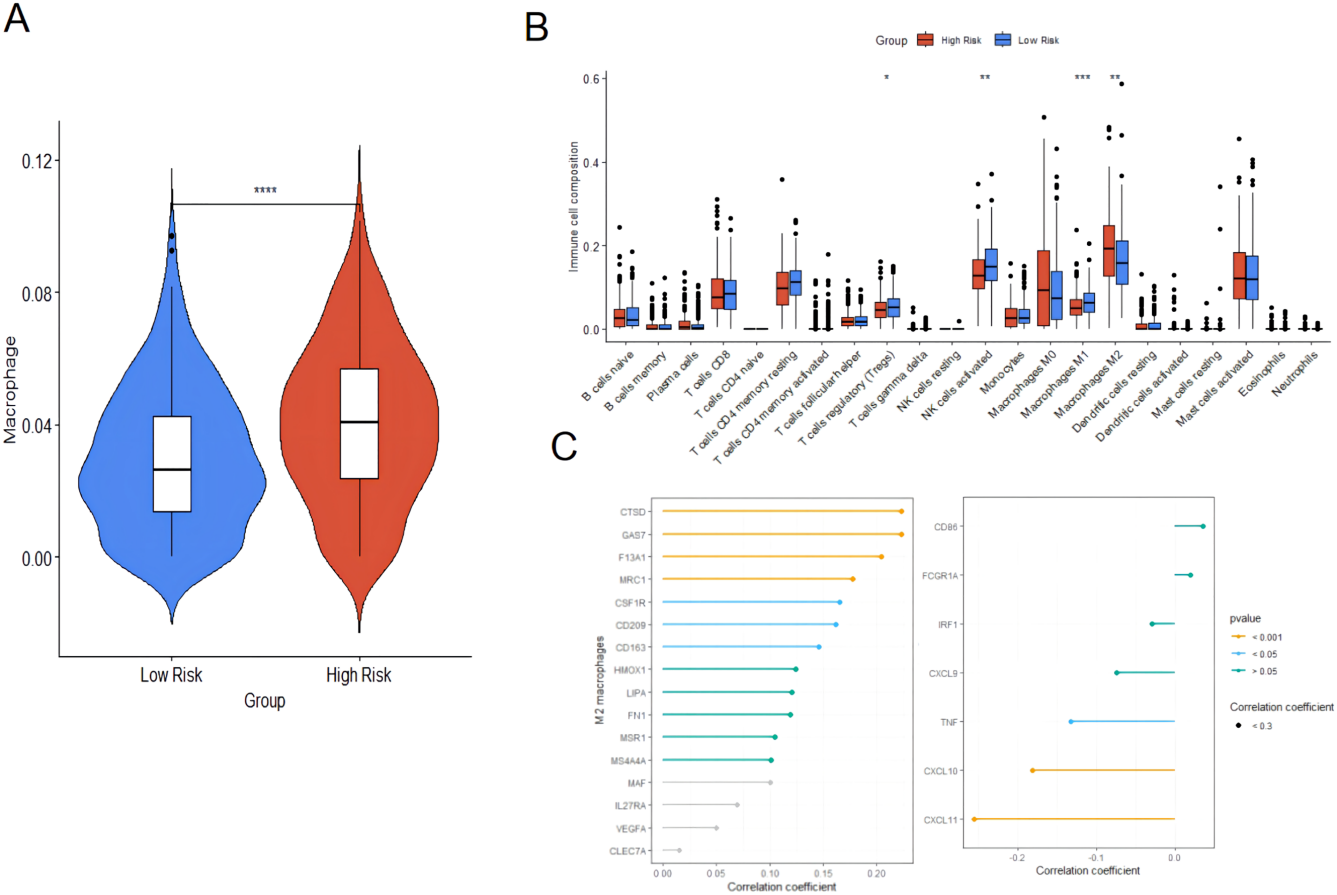
Immune cell infiltration analysis based on TIMER and immune cell infiltration analysis between high-risk group and low-risk group. **(A)** Immune cell infiltration analysis based on TIMER. **(B)** CIBERSORT algorithm; **(C)** Correlation between risk score and expression of M2 macrophage and M1 macrophage markers. * p<0.05, ** p<0.01, *** p<0.001, **** p<0.0001.

The TIMER database was used to evaluate the relationship between risk scores and immune cell infiltration since the tumor microenvironment is involved in tumor initiation, development, and metastasis (18). The prognosis of tumor patients with a high proportion of M2 macrophages infiltrating is poor, which may be due to the fact that M2 macrophages are conducive to the survival and proliferation of tumor cells by secreting some growth factors and inhibiting immune responses (19). The results revealed that the proportion of macrophage infiltration in the high-risk group was significantly higher than that in the low-risk group (**Figure 10A**), suggesting that macrophages might involved in the occurrence and development of ovarian cancer. Subsequently, the CIBERSORT algorithm used to evaluate the infiltration of 22 types of immune cells, including M1 and M2 macrophages showed that the content of M2 macrophages was significantly increased in the high-risk group of ovarian cancer. In contrast, In intra-group comparisons of M2 macrophage content, it was found that the content in the high-risk group was significantly higher than that in the low-risk group; in intra-group comparisons of M1 macrophage content, it was found that the content in the high-risk group was lower than that in the low-risk group (**Figure 10B**). Macrophage marker expression and risk score showed that the latter was positively correlated with the expression of M2 macrophage markers, and negatively correlated with the expression of M1 macrophage markers (**Figure 10C**). This result suggested that the high infiltration of M2 macrophages in patients with high ovarian cancer risk might be associated with poor prognosis.

**Figure 11:**
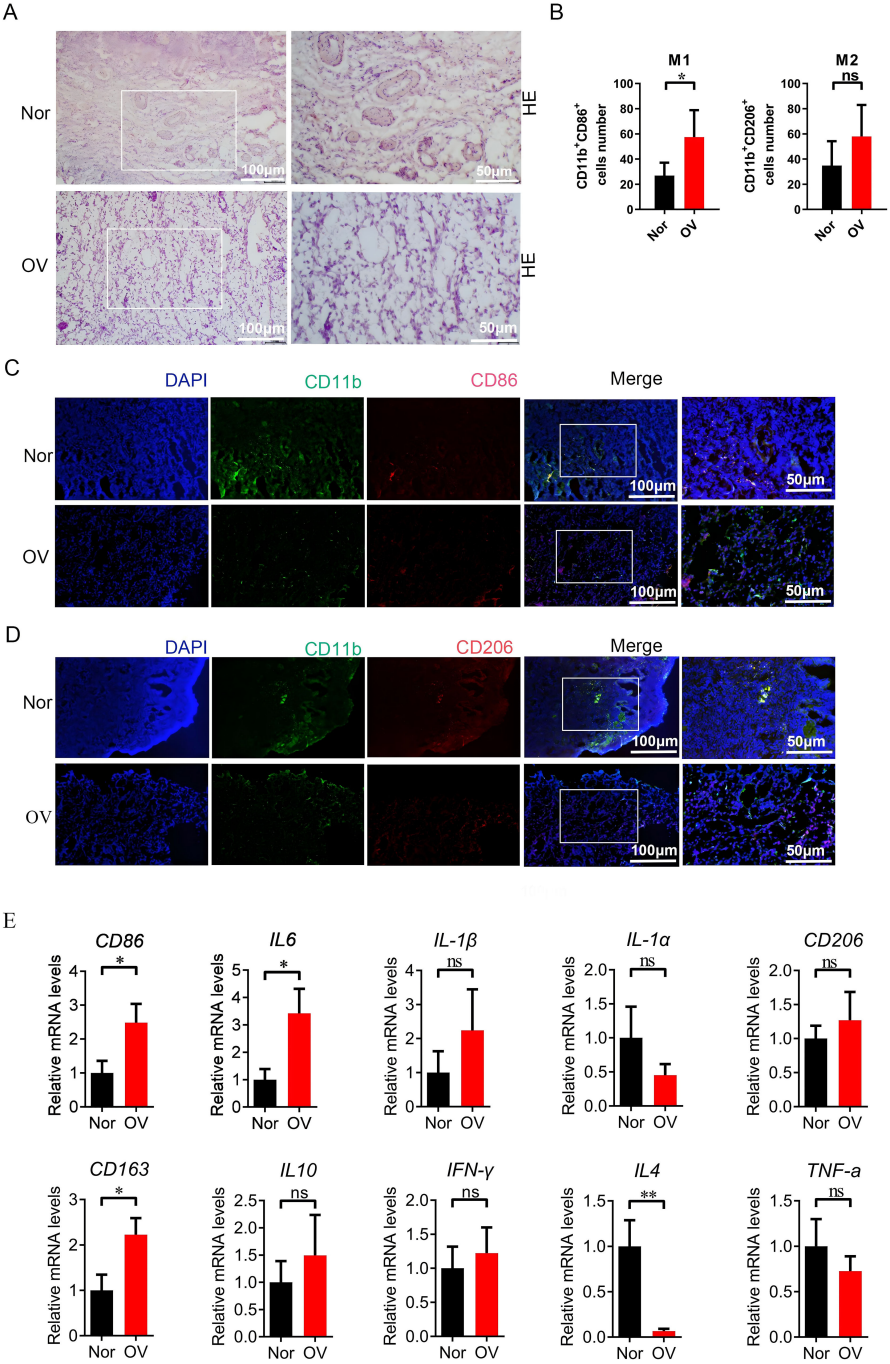
Compared with the Nor group, the M1/M2 macrophages in the ovarian tissue of the OV group were activated. **(A)** HE staining of ovarian tissues in the Nor group and the OV group; **(B–D)** Immunofluorescence double staining marked the changes in positive cells of M1 (CD11b^+^CD86^+^) and M2 macrophages (CD11b^+^CD206^+^) in ovarian tissues; **(E)** qPCR analysis of the relative mRNA expression of inflammatory factor genes related to M1 and M2 macrophages in the ovarian (8 samples in the Nor group and 8 samples in the OV group). * p<0.05, ** p<0.01, ns p>0.05.

We compared the macrophage activation analysis in normal ovarian tissue and ovarian cancer tissue, and performed HE staining on normal ovarian tissue (Nor group) and ovarian cancer tissue (OV group) to verify the histopathological changes in the samples of the two groups in this study. The results showed that the ovarian morphology, oocytes and follicle structure were normal in the ovarian tissue of the healthy group, while complete oocytes were not present in the ovarian cancer tissue, and tumor cell infiltration and disordered cell distribution were present (**Figure 11A**).The samples of Nor group and OV group were further labeled by immunofluorescence. In ovarian cancer, many studies have reported CD86 and CD206 as markers of M1 and M2 macrophage expression (20, 21). M1 type (CD11b^+^CD86^+^) and M2 type macrophages (CD11b^+^CD206^+^) showed a significantly growing trend in the OV group compared with the Nor group. A significant difference in the number of phagocytes was observed; the increase of phagocyte in OV group was significantly higher than that in Nor group (*P* < 0.05) (**Figures 11B–D**). The mRNA expression of the M1-related inflammatory factors CD86, IL6, and IL1-β was significantly increased in the OV group than in the Nor group. The mRNA expression of the M2-related inflammatory factors CD206, CD163, and IL10 were also significantly increased, and the variation trend of CD163 has significant difference. In this study, the expression of IL4 in ovarian cancer tissues was significantly reduced compared with the Nor group, suggesting that the prognosis of ovarian cancer was poor. The expression of the inflammatory factor IFN-γ was up-regulated, while that of TNF-α was down-regulated in the OV group (**Figure 11E**). These results demonstrated that the M1 and M2 macrophages in the ovarian tissue of the OV group were significantly activated than in the Nor group, and the polarity change of tumor-associated macrophages might provide a research reference for the prognosis and treatment of ovarian cancer.

### Correlation analysis between mitochondrial risk score and response to immunotherapy

**Figure 12:**
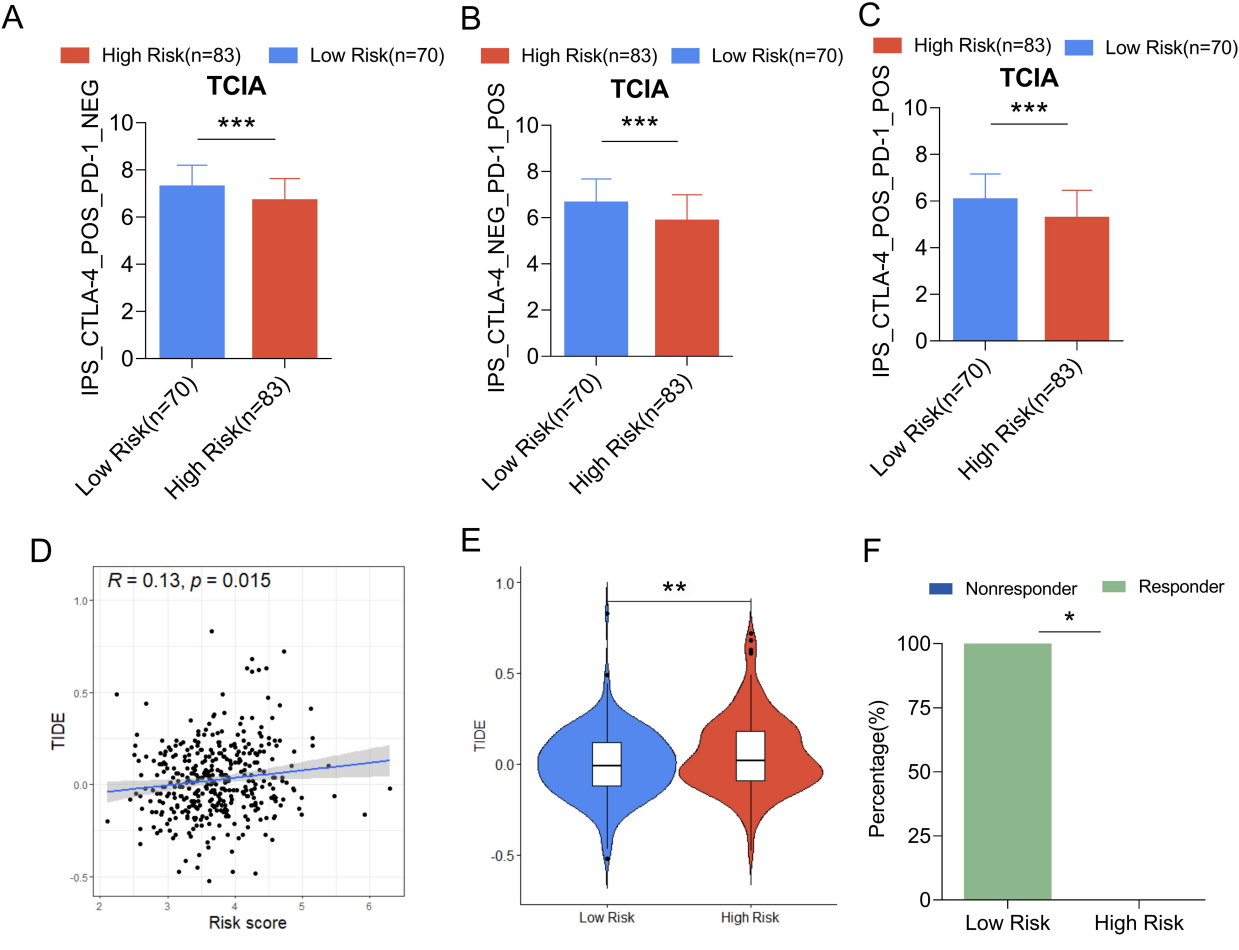
Comparison of IPS scores between high and low risk groups in the training set and predictive ability of mitochondrial risk score for immunotherapy response based on TIDE algorithm. **(A)** Comparison of IPS scores between the two groups of patients treated with CTLA-4 inhibitors; **(B)** Comparison of IPS scores between the two groups of patients treated with PD-1 inhibitors; **(C)** Comparison of IPS scores between the two groups of patients treated with CTLA-4 inhibitors combined with PD-1 inhibitors. **(D)** Linear analysis of risk score and TIDE score; **(E)** Comparison of TIDE scores between high-risk and low-risk groups; **(F)** Comparison of immunotherapy efficacy between high-risk and low-risk groups. P value * P<0.05, **P < 0.01, *** p< 0.001.

IPS expression in the low-risk group receiving CTLA-4, PD-1, and CTLA-4 combined with PD-1 treatment was significantly higher than that of the high-risk group (**Figures 12A–C**), suggesting ovarian cancer. Patients in the low-risk group might benefit from immune checkpoint therapy. The TIDE algorithm was further used to evaluate the ability of mitochondria-related risk scores to predict the response to immunotherapy in ovarian cancer, showing that the risk score was significantly positively correlated with the TIDE score (**Figure 12D**). The TIDE score of the high-risk group was significantly higher than that of the low-risk group (**Figure 12E**). The response rate to immunotherapy (46.5%) in the high-risk group was significantly higher. Lower than that in the low-risk group (58.2%) (**Figure 12F**), indicating that patients in the low-risk group of ovarian cancer respond better to immunotherapy.

**Figure 13:**
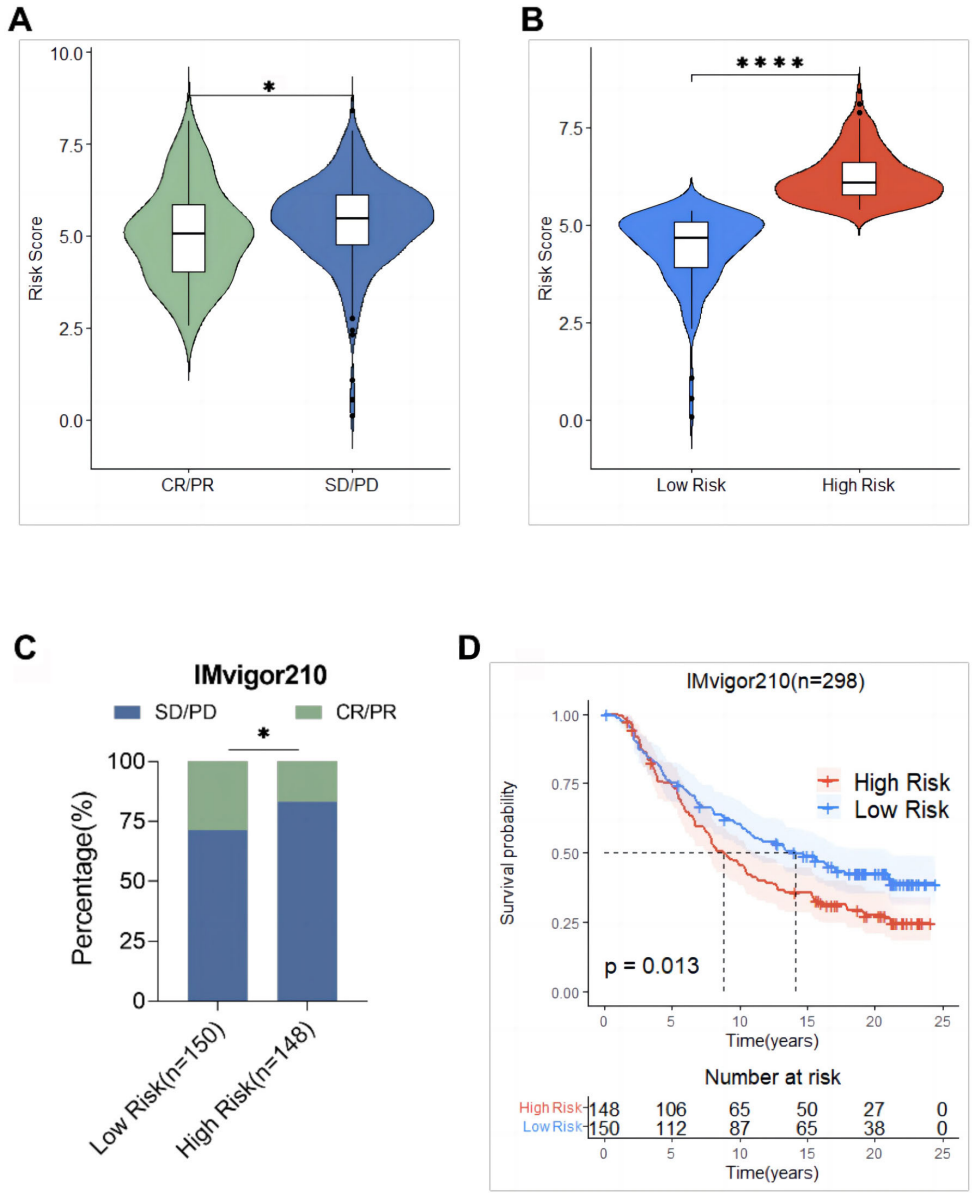
Validation of mitochondrial risk scores based on the IMvigor210 cohort predictive power of immunotherapy response. **(A)** Comparison of risk scores between the response (CR/PR) group and the non-response (SD/PD) group; **(B)** Comparison of risk scores between the high-risk group and the low-risk group; **(C)** Comparison of immunotherapy efficacy between the high-risk group and the low-risk group; **(D)** Comparison of overall survival between the high-risk group and the low-risk group. * p<0.05, **** p<0.0001.

The mitochondria-related risk scores in the IMvigor210 cohort were calculated and the relationship between risk scores and the efficacy of immunotherapy was assessed to verify the predictive ability of mitochondria-related risk scores on the immunotherapy response, based on the risk score Equation 1. The results showed that the risk score of the SD/PD group was significantly higher than that of the CR/PR group (**Figure 13A**). Subsequently, the IMvigor210 cohort was divided into a high-risk group (148 cases) and a low-risk group (150 cases) using the median risk score 5.35 as the cut-off value. The risk score of patients in the high-risk group was significantly higher than that in the lower-risk group. The response rate to immunotherapy in patients in the high-risk group (16.9%) was significantly lower than that in the low-risk group (28.7%) (**Figures 13B, C**). The KM survival analysis of patients in the IMvigor210 cohort showed that the OS of patients in the high-risk group was significantly shorter than that of patients in the low-risk group (*P* = 0.013, **Figure 13D**).

### Prediction of the effect of the mitochondrial risk score on chemotherapy in ovarian cancer

**Figure 14:**
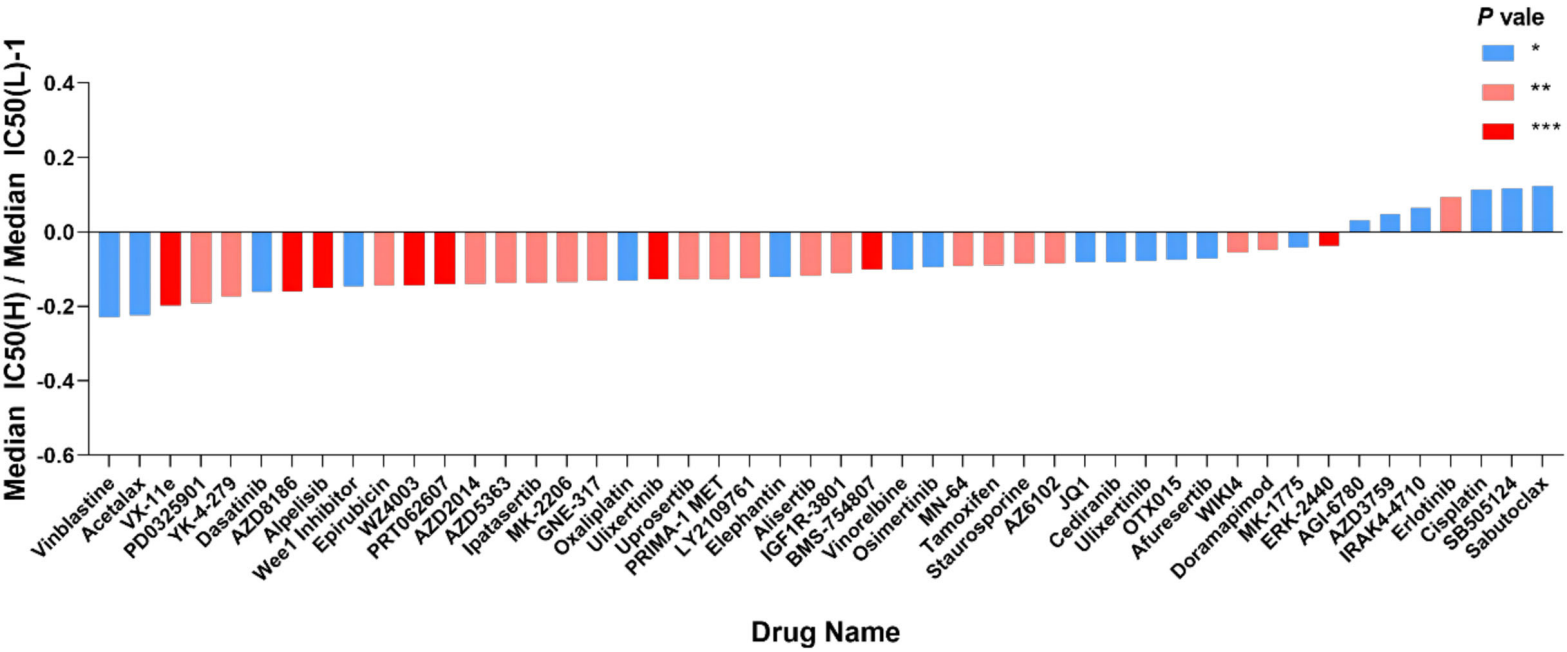
Value of risk score in predicting ovarian cancer chemotherapy treatment. The vertical axis represents the difference between the median IC50 of the high-risk group and the median IC50 of the low-risk group minus 1; the horizontal axis represents the names of 48 chemotherapy drugs. *P<0.05; **P<0.01; ***P<0.001. Vinblastine, Changchun alkaloid; Cisplatin, cisplatin; Erlotinib, erlotinib.

The IC50 values of 198 chemotherapeutic drugs used to combat ovarian cancer were predicted based on the GDSC database to explore the possibility of using the mitochondrial risk score to predict a personalized medication for ovarian cancer patients. Drug sensitivity analysis showed that patients in the high-risk group of ovarian cancer were remarkably sensitive to chemotherapeutic drugs such as vinblastine, Acetalax, VX-11e and PD-0325901, while patients in the low-risk group of ovarian cancer were significantly more sensitive to Sabutoclax, SB-505124, cisplatin and erlotinib, suggesting that mitochondrial risk scores provided a reliable reference for clinical treatment (**Figure 14**).

## Discussion

This study used public databases to demonstrate that the mitochondrial gene model could predict the prognosis of ovarian cancer patients with several unique advantages. First, the constructed risk scoring model had a strong predictive ability for the prognosis of ovarian cancer and is an independent prognostic factor affecting the OS of patients. Second, it provided a simple and feasible method to distinguish the belonging of the patient to the high-risk group or the low-risk group. In addition, the constructed risk scoring model could be used to predict the response to immunotherapy and chemotherapeutic drugs, as well as for the evaluation of immune cell infiltration, providing personalized management and treatment for ovarian cancer patients. The risk scoring model constructed based on 12 genes including RPL23 provided important evidence for further understanding the mechanism of the occurrence and development of ovarian cancer.

In this study, 12 prognostic characteristic genes were identified. RPL23 is a protein composed of 60S ribosomal subunits and involved in a variety of physiological and pathological processes including cell proliferation, apoptosis and cell cycle arrest. It affects the occurrence and development of cancer by specifically binding to mouse double minute 2 (MDM2) to affect the MDM2/p53 signaling pathway (22), exerting different biological effects in different tumors. For example, it is upregulated in hepatocellular carcinoma, gastric cancer, and pancreatic cancer, while it is downregulated in nasopharyngeal carcinoma cell lines and colorectal cancer cell lines. PKM2 is a rate-limiting enzyme in glycolysis and a regulator of tumor metabolism. It is overexpressed in several tumors, promoting their proliferation and metastasis (23). Patients with ovarian cancer and high PKM2 protein expression have shorter OS compared to those with low protein expression (24). MRPS12 is a potential oncogene for ovarian cancer, being a potential prognostic biomarker (25). The results of this study were consistent with the above conclusions, although further research is needed to explore the mechanism of MRPS12 in the occurrence and development of ovarian cancer. HPDL is a mitochondrial gene that encodes a 4-hydroxyphenylpyruvate dioxygenase-like protein. It is associated with several diseases including breast cancer. Its overexpression promotes the malignant progression of pancreatic ductal adenocarcinoma cells (26). The protein encoded by the MRPL14 gene promotes the biogenesis of mitochondrial large ribosomal subunits and mitochondrial translation (27). It is highly expressed in thyroid cancer and it is a potential oncogene (28). The protein encoded by COA6 is mainly located in the inner membrane of mitochondria and is mainly involved in the formation of cytochrome C oxidase (29). It is significantly upregulated in lung adenocarcinoma and is associated with poor prognosis (30). ACSM1 is highly expressed in prostate cancer and promotes its metastasis through the extracellular matrix-receptor interaction signaling pathway (31). However, it is poorly expressed in ovarian cancer (32). The ALDH1L1 gene, located on chromosome 3q21.3, encodes a protein that belongs to the aldehyde dehydrogenase family. Its loss of function or expression is associated with decreased apoptosis, increased cell motility, and cancer progression (33). ALDH1L1 mRNA and protein expression is significantly reduced in hepatocellular carcinoma, and the low expression of the protein is a potential prognostic marker for hepatocellular carcinoma (34). RNF144B, located on the mitochondrial membrane, negatively regulates apoptosis and ubiquitin-dependent protein catabolism (35). It promotes the proliferation, migration, and invasion of ovarian cancer cells (36). CAPN10, a member of the mitochondrial calpain system, promotes caspase-independent programmed cell death by mediating apoptosis-inducing factors. Its expression is associated with insulin-stimulated glucose uptake and type 2 diabetes (37). This gene is regulated by the GAEC1 gene and promotes the progression of esophageal squamous cell carcinoma (38). In the present study, RPL23, FGFR1OP2, CAPN10, ALDH1L1 and ACSM1 were significantly downregulated in ovarian cancer, while the up-regulation of RPL23, FGFR1OP2, CAPN10 and ALDH1L1 was associated with poor prognosis in patients with ovarian cancer. PKM2, MRPS12, NDUFC2, HPDL, MRPL14, COA6 and RNF144B were significantly upregulated in ovarian cancer, but the down-regulation of NDUFC2, HPDL, MRPL14, COA6 and RNF144B was associated with poor prognosis in patients with ovarian cancer. The expressions of the twelve genes in ovarian cancer cells were detected and the expected consistent results were obtained.

Biomarkers such as RPL23, MRPS12, and ALDH1L1 have been used to predict the prognosis of ovarian cancer, but most studies focus on the prognostic role of a single biomarker (25, 39, 40). However, prognostic models constructed using multiple genes are more comprehensive and effective in different malignancies than prognostic models constructed using a single gene. For example, Ye et al. (41) constructed an ovarian cancer prognostic model based on histone acetylation-related genes, and Qi et al. (42) constructed an ovarian cancer prognostic model related to ferroptosis, while in this study an ovarian cancer prognostic prediction model was constructed based on mitochondrial genes.

Accumulating somatic mutations lead to the occurrence of cancer and promote the expression of neoantigens (43). TMB has a specific value in predicting the prognosis of patients with liver cancer and gastric cancer (44, 45), suggesting that it might become a new prognostic prediction marker. The results of this study are consistent with those in the above-mentioned studies. The mitochondria-related risk score was significantly negatively correlated with TMB, and the response rate to immunotherapy in ovarian cancer patients in the low-risk group was significantly higher than that in patients in the high-risk group. TMB and risk score differentiated the prognostic status of ovarian cancer patients.

Macrophage polarization is involved in the prognosis of ovarian cancer patients (46). The verification results of this study showed an increasing trend of both M1 and M2 macrophages in the OV group, and the number of M1 macrophages was significantly higher. The mRNA expression of the M1-related inflammatory factors CD86, IL6, and IL1-β was significantly increased in the OV group, that of the M2-related inflammatory factors CD206, CD163, and IL10 was also significantly increased. There are research reports that IL-4 has anti-tumor effects (47, 48), and its expression has important reference value for judging the malignancy of ovarian cancer and predicting prognosis. In this study the expression of the macrophage regulatory gene IL4 in the OV group was significantly down-regulated, suggesting that the prognosis of ovarian cancer was poor. The inflammatory factor IFN-γ was significantly up-regulated, and that of TNF-α was significantly down-regulated. The results showed that both M1 and M2 macrophages in the ovarian tissue of the OV group were significantly activated, representing a reference for the investigation of polarity changes in tumor-associated macrophages in the prognosis and treatment of ovarian cancer. In addition, M1 macrophages were prevalent in patients in the low-risk group, while M2 macrophages were prevalent in patients in the high-risk group. The expression of M2 macrophage markers in the high-risk group was significantly negatively correlated with the risk score, suggesting the presence of the immunosuppressive cells M2 macrophages in the high-risk group, creating an immunosuppressive microenvironment that inhibited the eradication of tumor cells mediated by the immune system. As a result, the prognosis of patients in the high-risk group of ovarian cancer was poor.

Tumor cells activate immune checkpoint pathways with different immunosuppressive functions (49). Immune checkpoint inhibitors have certain efficacy in the treatment of gynecological malignancies including ovarian cancer. The use of the TCIA database revealed that the IPS in the low-risk group was significantly higher than that in the high-risk group, suggesting that immune checkpoint therapy might be beneficial in patients in the low-risk group. In addition, the mitochondria-related risk score in the training set was positively correlated with the TIDE score, suggesting that the lower the risk score, the more likely immunotherapy is beneficial. Several reports show that cross-tumor information can be used to predict the effect of immunotherapy (44, 50). The ability to predict the immunotherapy response by mitochondria-related risk scores was verified using the data of 298 urothelial cancer patients from the IMvigor210 cohort, which showed that the response rate to immunotherapy in the high-risk group (16.9%) was significantly lower than that in the low-risk group (28.7%), and the OS of patients in the high-risk group was significantly shorter than that of patients in the low-risk group. This suggested that the mitochondria-related risk score could be used as a powerful indicator to predict the response to immunotherapy in ovarian cancer.

Risk scores help the identification of therapeutic drugs that are beneficial for ovarian cancer patients. In this study, vinblastine, Acetalax, VX-11e, and PD-0325901 were more effective in the high-risk group, while Sabutoclax, SB-505124, cisplatin, and erlotinib were more effective in the low-risk group.

This study has some limitations: ① the constructed risk scoring model needs to be further verified using prospective clinical data; ② the mechanism and role between prognostic genes and mitochondrial dysfunction still need to be explored.

In conclusion, a prognostic risk score model for ovarian cancer patients was constructed in this study based on PL23, PKM2, MRPS12, NDUFC2, HPDL, MRPL14, COA6, FGFR1OP2, RNF144B, CAPN10, ALDH1L1, and ACSM1. The risk score was not only highly correlated with macrophage infiltration in ovarian cancer patients but was also a good predictor of the response to immunotherapy. In terms of drug sensitivity, patients in the high-risk group were more sensitive to vinblastine, Acetalax, VX-11e, and PD-0325901, while patients in the low-risk group were more sensitive to Sabutoclax, SB-505124, cisplatin, and erlotinib. Thus, the mitochondrial-related risk model might become a reliable prognostic biomarker for the personalized treatment of ovarian cancer patients.

## Data Availability

The original contributions presented in the study are included in the article/**Supplementary Material**. Further inquiries can be directed to the corresponding author.
