# Supplementary material for: Explore the expression of mitochondria-related genes to construct prognostic risk model for ovarian cancer and validate it, so as to provide optimized treatment for ovarian cancer
Source: Front Immunol. 2024 Oct 16;15:1458264. doi: 10.3389/fimmu.2024.1458264 (PMC11521951; doi:10.3389/fimmu.2024.1458264)
Supplement: Supplementary file 1 [file Table1.docx]

**Table1 : Demographic and clinical characteristics of the patients.**

| **Number** | ***Age*** | ***FIGO staging*** |
| --- | --- | --- |
| patient1 | 30 | IVB |
| Patient2 | 52 | IVB |
| Patient3 | 44 | IIIC |
| Patient4 | 67 | IVB |
| Patient5 | 57 | IVB |
| Patient6 | 50 | IVB |
| Patient7 | 50 | IVB |
| Patient8 | 54 | IVB |
| Patient9 | 50 | IVB |
| Patient10 | 62 | IVB |
| patient11 | 43 | IIIC |
| patient12 | 61 | IVA |
| patient13 | 58 | IVA |
| patient14 | 38 | IVA |
| patient15 | 53 | IVA |
| patient16 | 60 | IVA |

**Table2 : Primers for real time-qPCR**

| *CD86* | *F CTGCTCATCTATACACGGTTACC* |
| --- | --- |
|  | *R GGAAACGTCGTACAGTTCTGTG* |
| *IL6* | *F ACTCACCTCTTCAGAACGAATTG* |
|  | *R CCATCTTTGGAAGGTTCAGGTTG* |
| *IL-1β* | *F AGCTACGAATCTCCGACCAC* |
|  | *R CGTTATCCCATGTGTCGAAGAA* |
| *IL-1α* | *F TGGTAGTAGCAACCAACGGGA* |
|  | *R ACTTTGATTGAGGGCGTCATTC* |
| *CD206* | *F GGGTTGCTATCACTCTCTATGC* |
|  | *R TTTCTTGTCTGTTGCCGTAGTT* |
| *CD163* | *F GACGCATTTGGATGGATCATGT* |
|  | *R CCCACCGTCCTTGGAATTTGA* |
| *IL10* | *F GACTTTAAGGGTTACCTGGGTTG* |
|  | *R TCACATGCGCCTTGATGTCTG* |
| *IFN-γ* | *F* *TCGGTAACTGACTTGAATGTCCA* |
|  | *R* *TCGCTTCCCTGTTTTAGCTGC* |
| *IL4* | *F ATGGGTCTCACCTCCCAACT* |
|  | *R GATGTCTGTTACGGTCAACTCG* |
| *TNF-α* | *F* *CCTCTCTCTAATCAGCCCTCTG* |
|  | *R* *GAGGACCTGGGAGTAGATGAG* |
| *PL23* | *F GGGTGTTCCGAACCAACTTCT* |
|  | *R GCCACGGGCACGTTATAGAT* |
| *PKM2* | *F AAGGGTGTGAACCTTCCTGG* |
|  | *R GCTCGACCCCAAACTTCAGA* |
| *MRPS12* | *F ACTCAGCCAATCGCAAGTG* |
|  | *R CGCCCTCCACAAGGACAAT* |
| *NDUFC2* | *F CGGCCTGATTGATAACCTAATCC* |
|  | *R AAGCTGGCGATGCAAACCA* |
| *HPDL* | *F TGGGGCATACTACCAGCAG* |
|  | *R AGATTGCTCCTGTACGGACTG* |
| *MRPL14* | *F GAAGAAAAAGGCGCTCATTGTG* |
|  | *R ACGTTGTTGGAGTCGAATCTG* |
| *COA6* | *F AAAGAGGCGCAGCGTATCTC* |
|  | *R TTGCATAATGCCGGGCACTG* |
| *FGFR1OP2* | *F CCACGTTAGTTATGGGAATCCAG* |
|  | *R CAAGGCCGACTGATGTTCTTC* |
| *RNF144B* | *F GACAGTCAGCCTATTGTCCTGC* |
|  | *R TCATTGCGTTCGATATAAACCCG* |
| *CAPN10* | *F GGTCTCAGAACCGAGTGAGGT* |
|  | *R CCACGAAGTATGACTGTCACC* |
| *ALDH1L1* | *F TCCAGACCTTCCGCTACTTTG* |
|  | *R CAGGGGATAGTTCCAGGGGAT* |
| *ACSM1* | *F CGTGTAGCCAACGTCTTCACA* |
|  | *R AAGGGCATCTATGGTCACAATG* |

**Table3 :Treatment status of 8 patient samples**

| Sample number | Final Diagnosis  (FIGO Staging) | Surgical modality | Postoperative chemotherapy regimen and dose |
| --- | --- | --- | --- |
| 1 | IVB | Total hysterectomy with bilateral adnexect- omy, partial rectal, sigmoid colon, left colon, greater omentum, appendix, pelvic and abdominal lymph node dissection, presacral lymph node dissection, diaphrag- matic peritonectomy, peritoneal stripping of the bladder surface, resection of lesions in the right colonic groove, resection of lesions in the porta hepatis, resection of the hepatoduodenal ligament, placement of a venous infusion port | Paclitaxel 350mg, Carboplatin700mg |
| 2 | IVB | Total hysterectomy with bilateral adnexect- omy, resection of liver metastases, splenect- omy, right diaphragmatic resection, after resection of the greater omentum, para-aortic lymph node dissection, pelvic lymph node dissection, uterosacral ligament resection, placement of a venous infusion port | Paclitaxel 350mg, Carboplatin700mg |
| 3 | IVB | Total hysterectomy with bilateral adnexect- omy, resection of liver metastases, splenect- omy, right diaphragmatic resection, after resection of the greater omentum, para-aortic lymph node dissection, pelvic lymph node dissection, uterosacral ligament resection, placement of a venous infusion port | Paclitaxel 150mg, Carboplatin150mg |
| 4 | IVB | Bilateral ovarian tumors, lesions on the greater omentum, surface lesions on the rectum, clearance of pelvic and abdominal lesions, eradication of tumor cells | Paclitaxel 350mg, Carboplatin450mg |
| 5 | IVB | Total hysterectomy with bilateral adnexect- omy, hepatic metastasis, splenectomy, right diaphragmatic resection, after greater omentum resection, para-aortic lymph node dissection, pelvic lymph node dissection, uterosacral excavation, placement of a venous infusion port | Paclitaxel 400mg, Carboplatin550mg |
| 6 | IVB | Uterine adnexa, greater omentum, hepatic ligaments, transverse colon, pelvic and abdominal lymph nodes, appendix, diaphragm, bladder | Dorou 40mg, Carboplatin 500mg |
| 7 | IVB | Total hysterectomy with bilateral adnexect- omy, hepatic metastasis, splenectomy, right diaphragmatic resection, after greater omentum resection, para-aortic lymph node dissection, pelvic lymph node dissection, uterosacral excavation, placement of a venous infusion port | Paclitaxel 350mg, Carboplatin 450mg |
| 8 | IIIC | Total hysterectomy with bilateral adnexect- omy, greater omentum, pelvic and abdominal lymph nodes, transverse colon, rectum. | Paclitaxel 350mg, Carboplatin 450mg |
